# White Matter Alterations Are More Robust Than Gray Matter Differences in Tinnitus and Hearing Loss: An MRI Study of Military Personnel

**DOI:** 10.1007/s10162-026-01052-0

**Published:** 2026-05-13

**Authors:** Emilia Kaniewska, Gibbeum Kim, Rafay A. Khan, Yihsin Tai, Caterina M. Willson, Carlos Esquivel, Paul M. Sherman, Fatima T. Husain

**Affiliations:** 1 Department of Speech and Hearing Science, University of Illinois Urbana-Champaign, 901 S. Sixth Street, Champaign, IL 61820 USA; 2 Beckman Institute for Advanced Science and Technology, University of Illinois at Urbana-Champaign, Champaign, IL 61801 USA; 3 Neuroscience Program, University of Illinois at Urbana-Champaign, Champaign, IL 61801 USA; 4 Department of Speech Pathology and Audiology, Ball State University, Muncie, IN 47306 USA; 5 Department of Defense Hearing Center of Excellence, San Antonio- Lackland, TX 78236 USA; 6 Department of Otolaryngology, UT Health San Antonio, San Antonio, TX 78249 USA; 7 United States Air Force , Arlington County, VA USA; 8 American College of Radiology, Preston White Dr, Reston, VA 20191 USA; 9 Aerospace Medical Association, North Palm Beach, FL USA

**Keywords:** Tinnitus, Hearing loss, Gray matter, White matter, Voxel-based morphometry, Diffusion tensor imaging

## Abstract

**Purpose:**

Tinnitus and hearing loss are the most prevalent service-related auditory disabilities among American veterans. Previous studies have examined gray matter or white matter alterations in tinnitus and hearing loss relative to healthy controls, but typically in isolation, and none of them have focused specifically on a military-affiliated population.

**Methods:**

We employed voxel-based morphometry to assess gray matter differences and diffusion tensor imaging to evaluate white matter integrity in tinnitus and/or hearing loss compared to controls in a sample of 68 military-affiliated adults (56 men, 10 women, two identifying as ‘other’ gender). Additionally, we conducted an exploratory, hypothesis-generating effect size analysis to describe the magnitude of group differences.

**Results:**

Combined tinnitus and hearing loss was associated with decreased gray matter volume in the thalamus. White matter integrity was reduced in the forceps minor, right superior longitudinal fasciculus, and left inferior longitudinal fasciculus. Hearing loss alone was associated with white matter orientation changes in the right anterior thalamic radiation, left superior longitudinal fasciculus, and right corticospinal tract. Larger effect sizes were noted in white matter comparisons across tinnitus, hearing loss, and combined conditions, suggesting that white matter differences may be of greater magnitude than gray matter alterations.

**Conclusion:**

These findings advance the understanding of the neural correlates of tinnitus and hearing loss in the military population, an understudied group with a high prevalence of tinnitus and hearing loss. They also present preliminary effect size estimates that may inform future studies on the neural correlates of tinnitus and hearing loss, indicating a larger overall magnitude in white matter and more subtle contributions from gray matter.

**Supplementary Information:**

The online version contains supplementary material available at 10.1007/s10162-026-01052-0.

## Introduction

Tinnitus, often described as a phantom sound, is the perception of sound without any external source [1, 2]. Approximately 5–15% of the American population experiences tinnitus [3]. While hearing loss is commonly comorbid with tinnitus, with 90% of individuals with tinnitus also experiencing hearing loss, only 30–40% of individuals with hearing loss report tinnitus [4–6]. This asymmetry suggests that hearing loss alone does not fully account for the presence of tinnitus, indicating that additional factors such as neuroplastic changes may contribute to its development and persistence [4, 7–9]. Beyond perceiving the sounds of tinnitus, people living with tinnitus often experience additional challenges such as anxiety, depression, sleep disturbance, concentration and communication challenges, and reduced quality of life [4]. These difficulties highlight the profound impact of tinnitus and the need for continued research, particularly in populations where tinnitus is highly prevalent and potentially more burdensome, such as among military personnel, who are frequently exposed to impulse noise, blast trauma, and other service-related risk factors, including chemical agents [10, 11]. Despite the high prevalence of tinnitus and hearing loss in military cohorts, no neuroanatomical studies, focused on both gray and white matter, have directly examined this population.

To better understand mechanisms beyond primary auditory pathway involvement that contribute to tinnitus, it is essential to investigate neuroanatomical changes in the brain. Structural imaging techniques such as voxel-based morphometry (VBM) and diffusion tensor imaging (DTI) have been used to identify brain-level differences between individuals with tinnitus and those without (for a review, see [12]). These brain-level differences may play a role in the development and persistence of tinnitus, even in individuals without marked hearing loss. VBM identifies changes in gray matter (GM) volume, while DTI reveals disruptions in white matter integrity. Studying both allows researchers to pinpoint where structural alterations occur and how the structural communication pathways may be compromised at the brain level. This dual approach improves our ability to detect consistent neuroanatomical patterns, distinguish tinnitus-related changes from those due to hearing loss, and inform future efforts to develop imaging-based markers for diagnosis or intervention.

In the following sections, we review the literature on the use of brain imaging techniques, specifically VBM and DTI, focusing on findings from studies that have used these methods to examine tinnitus and hearing loss.

### VBM

Numerous studies have investigated GM differences in individuals with tinnitus and those without to uncover the underlying neural mechanisms of tinnitus [13]. VBM is a widely used neuroimaging technique that uses T1-weighted magnetic resonance imaging (MRI) scans to assess GM volume at the voxel level. This can be used to calculate the total volume of the whole brain, or of targeted substructures, and can then be compared across participant groups. This technique is particularly valuable for detecting changes that occur over time due to neuroplasticity and brain reorganization [14]. In individuals with tinnitus and/or hearing loss, decreased GM volume may reflect a lower proportion of GM tissue in a given region, which may indicate structural adaptation, reduced processing demand, or altered anatomical connectivity [15]. These changes may suggest that the brain has reorganized in response to auditory dysfunction, and VBM offers a systematic, whole-brain approach to localizing and quantifying such adaptations across individuals and groups.

Previous VBM studies on tinnitus have produced varied results, as highlighted in a review [12]. The first VBM study on tinnitus [16] identified a decrease in GM in the subcallosal area in individuals with tinnitus, suggesting diminished inhibitory feedback, which may lead to the failure of canceling out the noise and constant sound perception in the auditory cortex. However, subsequent attempts to replicate these findings, such as the study by Landgrebe et al. [17], revealed GM reduction in other brain regions. These regions include the right inferior colliculus and the left hippocampus, which are associated with auditory processing and memory, respectively, suggesting that tinnitus may involve both sensory and cognitive-emotional components. Since then, GM decreases in the tinnitus population have been reported in both auditory processing regions [14, 18, 19] and the limbic system [20, 21], indicating the involvement of both sensory and emotional processing regions. A meta-analysis of 17 resting-state voxel-based physiology (VBP) studies and eight VBM structural studies revealed decreases in the default mode network (DMN), particularly in the posterior cingulate cortex, cingulate gyrus, and precuneus [22]. These findings suggest that attentional and awareness-related processes significantly influence tinnitus perception. Additionally, some studies have reported null findings regarding tinnitus [15, 23–25], which may reflect limitations in the sensitivity of the VBM technique to subtle neuroanatomical changes or variability in sample characteristics across studies.

Hearing loss is a significant factor in VBM studies and often is associated with greater changes in brain structure than tinnitus [12]. Gray matter decreases associated with hearing loss are typically observed in auditory processing regions, such as the primary auditory cortex and inferior colliculus [14, 15]. Additionally, higher-level cognitive processing regions, including the prefrontal cortex, hippocampus, and posterior cingulate cortex, are affected particularly in cases of severe or long-term hearing loss [15, 18, 19, 26]. Interestingly, some studies suggest that tinnitus may mitigate hearing loss-related GM decline; Koops et al. [25] reported that when comparing age-matched and hearing-matched participants without tinnitus to those with tinnitus, the expected decreases in GM volume were absent in the tinnitus group. This suggests that tinnitus may help maintain GM volume in individuals with hearing loss [25]. Differences in findings from VBM studies may be attributed to several factors, including the heterogeneity of tinnitus etiology, small sample sizes, age differences, and the presence of additional hearing loss.

### DTI

DTI is a neuroimaging technique used to assess the microstructural integrity of white matter by measuring the movement of water molecules within tissue. It captures directional patterns of diffusion, shaped by the organization of axonal fibers, myelin, and cellular architecture. Fractional anisotropy (FA), a commonly used DTI metric, quantifies the degree to which water diffusion is directionally constrained. Higher FA values reflect greater fiber coherence, density, or myelination, while lower FA values may reflect reduced structural integrity. As such, FA provides a sensitive measure for detecting subtle microstructural changes in white matter pathways.

Although DTI studies of tinnitus and hearing loss have faced challenges and inconsistencies similar to those in the VBM studies, some commonalities have emerged. The most consistent findings point to white matter changes in tracts underlying the auditory cortex as well as in auditory-limbic and limbic pathways, primarily in individuals with tinnitus compared to healthy controls or hearing-matched participants without tinnitus [27–32]. Specific tracts showing altered microstructural integrity include the corpus callosum [29, 33–35], cingulum [34], and superior longitudinal fasciculus [36], with most studies reporting decreased FA in tinnitus compared to controls, suggesting reduced white matter integrity. Additionally, a systematic review of 20 DTI studies exploring the effect of hearing loss alone reported FA decrease in various auditory-related brain regions, including the corpus callosum, forceps major, left parahippocampal cingulum, and left superior thalamic radiation [37].

Building on earlier findings, a recent study by Xu et al. [38] using multi-shell DTI further supports the presence of white matter alterations in tinnitus. The authors reported changes in frontal and motor pathways, particularly in the forceps minor and right corticospinal tract. These disruptions were linked to reduced network efficiency and cognitive performance. Notably, the study found that white matter changes were more prominent than gray matter changes. Despite these extensive findings, no study has systematically compared GM and WM alterations in military personnel, leaving a critical gap in understanding service-related auditory neurotrauma.

### Study Aims

Our present study addresses the above gap. It represents a baseline investigation of the tinnitus-related and hearing-loss-related brain changes in military personnel without diagnosed psychiatric comorbidities, offering a clearer view of structural brain differences in a population with elevated auditory risk. By focusing on this group, the study aims to reduce etiological heterogeneity that complicates civilian research and to deepen our understanding of the neural mechanisms underlying tinnitus and its comorbid hearing loss.

Based on previous VBM and DTI tinnitus research in the general population [14, 16, 18, 19, 22, 34, 35, 38], we hypothesized decreased GM volume in regions involved in auditory and emotional processing, including the auditory cortex, hippocampus, and limbic structures, as well as reduced white matter integrity in associated tracts such as the corpus callosum, cingulum, and superior longitudinal fasciculus. However, given the service-related experiences of military personnel, we anticipated that the pattern or extent of these structural differences may differ from those observed in non-military populations.

## Methods

### Study Participants

Military-affiliated participants (*n* = 68) aged 21 to 64 years (mean = 43.34 years, SD = 10.23 years) were included in the study. All participants were able to provide informed consent and met MRI safety requirements. Persistent tinnitus was defined as being non-pulsatile and chronic tinnitus based on self-report. Participants were divided into two primary groups: control (CON, *n* = 28) and tinnitus (TIN, *n* = 40). Control participants reported no current or past experience of persistent tinnitus. Participants with current severe depression or anxiety, as measured by the BDI-II [39] and BAI [40], were excluded from the study. The BDI-II and BAI each range from 0 to 63, with scores above 29 (BDI-II) and 25 (BAI) indicating severe symptoms. In this sample, mean scores were low, no greater than three, suggesting minimal levels of depression and anxiety across groups. Sex-based and gender-based analyses were considered but not performed due to the male-dominated military sample and insufficient representation of other genders for meaningful subgroup analysis.

### Behavioral Data Collection and Analysis

Data were collected at the Defense Health Agency Hearing Center for Excellence (HCE) at Joint Base San Antonio, Lackland Air Force, Texas, under the IRB Protocol number M-10546.

**Fig. 1:**
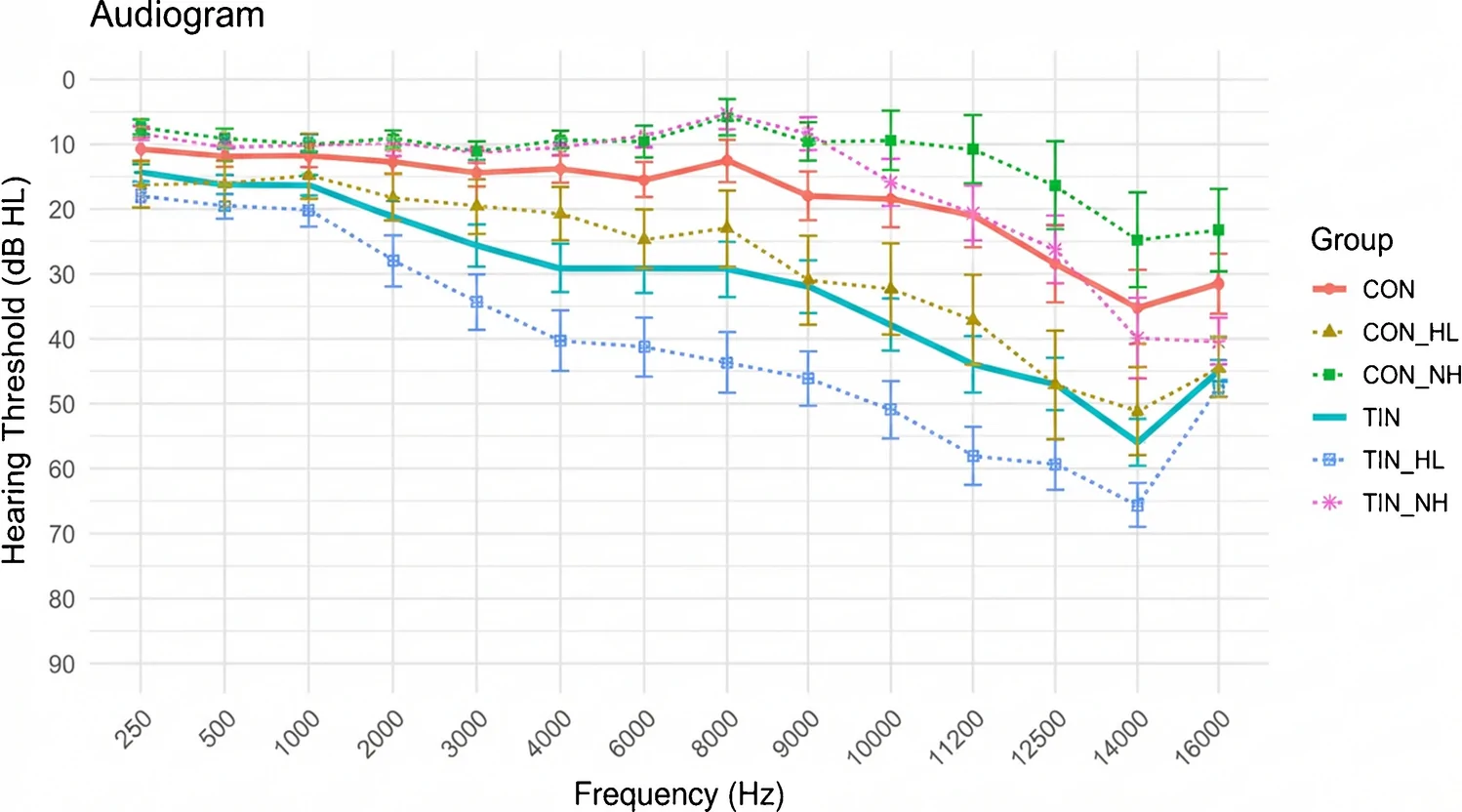
Bilateral averaged hearing thresholds for all groups and subgroups. Error bars represent the standard error of the mean

Pure-tone audiometry was conducted in a sound-treated environment to determine hearing status. Hearing loss was defined as a threshold exceeding 25 dB HL at any tested frequency between 0.25 and 8 kHz in either ear. Most hearing loss in this sample occurred in the higher frequencies (2–8 kHz), as shown in the group audiograms in Fig. 1. Therefore, group hearing status is further reported in Table 2 using the high-frequency pure-tone average (HFPTA). Detailed hearing procedures and results are reported in a separate study from our lab [41]. Each primary group was further subdivided according to hearing status: CON_NH_ (control with normal hearing), CON_HL_(control with hearing loss), TIN_NH_ (tinnitus with normal hearing), TIN_HL_ (tinnitus with hearing loss). Participants with tinnitus also completed the Tinnitus Functional Index (TFI: [42]) questionnaire to assess the severity of their tinnitus.

Behavioral data were analyzed using R statistics software (R Core Team, version 4.5.1 [43]). Normality and homogeneity of variance were confirmed using Shapiro-Wilk and Levene’s tests. Group-level comparisons between TIN and CON were conducted using independent *t*-tests for age, BAI, BDI-II, PTA, and HFPTA, with p-values adjusted for multiple comparisons. Gender differences were assessed using the chi-square test. To further explore demographic and behavioral variation across subgroups, one-way ANOVA was conducted for age, BAI, and BDI-II, followed by post-hoc Tukey HSD tests. Statistical comparisons of hearing thresholds were conducted at the level of the primary diagnostic groups (TIN vs CON), rather than across all four subgroups, because hearing status was defined categorically and is expected to produce significant differences in audiometric threshold design.

### VBM Data Preprocessing and Analysis

#### Preprocessing

Structural MRI data were acquired using a 3.0 T Siemens Prisma MRI scanner. High-resolution T1-weighted Magnetization Prepared Rapid Gradient Echo (MPRAGE) images were collected with the following parameters: orientation = sagittal, TR = 8.6 ms, TE = 4.00 ms, flip angle = 20°, voxel size = 1.2 × 1.1 × 7.0 mm^3^.

Raw DICOM data were converted to NIFTI format using MRIcron software (MRIcon, https://www.nitrc.org/projects/mricron/). Preprocessing was performed using the Computational Anatomy Toolbox (CAT12, https://neuro-jena.github.io/cat//index.html) implemented in SPM12 (https://www.fil.ion.ucl.ac.uk/spm/software/) running in MATLAB R2024b (The MathWorks Inc. 2024. MATLAB version R2024b. Natick, Massachusetts).

To account for individual anatomical variability, images were bias-corrected and spatially normalized to the standard template. Modulation was applied to preserve the total amount of signal after normalization. The normalized images were segmented into gray matter (GM), white matter (WM), and cerebrospinal fluid. Based on the study aim, only the GM images were smoothed using an 8-mm full-width-at-half-maximum (FWHM) Gaussian kernel, consistent with previous works in our lab [15, 19]. An 8-mm FWHM kernel was selected to balance sensitivity to small subcortical structures with comparability to prior tinnitus studies. Total intracranial volume (TIV) was also estimated for each participant.

#### Whole-Brain Analysis

A full factorial two-way ANOVA was conducted at the whole-brain level to examine the main effects of tinnitus and hearing loss. Post hoc *t*-tests were used to explore the interactions between the subgroups. Statistical significance was set at *p* < 0.05, corrected for multiple comparisons using family-wise error (FWE) correction. To explore potential subthreshold effects, the same analyses were also conducted at an uncorrected threshold (*p* < 0.001).

TIV and age were first included as covariates of no interest in all analyses to control for potential confounding effects. However, given concerns that age may overcorrect and obscure tinnitus-related effects, all analyses were repeated with TIV as the sole covariate. Both FWE-corrected and uncorrected thresholds were applied in the secondary analysis.

#### Region of Interest Analysis

A region of interest (ROI) analysis was conducted to focus on cortical and subcortical structures of the central auditory system, with additional regions in the medial and dorsolateral prefrontal cortex, to reduce the risk of Type I error. The mask from previous VBM studies conducted by Muhlau et al. [16] and Landgrebe et al. [17] was used, with the additional regions identified by Husain et al. [15]. The mask included 8-mm-radius spheres centered at the Montreal Neurological Institute (MNI) coordinates present in Table 1.

**Table 1:** The locations of the seeds used in the regions of interest (ROI) analysis for the VBM study

Region	MNI coordinates
Bilateral ventral and dorsal cochlear nuclei	± 10, −38, −45
Bilateral superior olivary complexes	± 13, −35, −41
Bilateral inferior colliculi	± 6, −33, −11
Bilateral medial geniculate nuclei	± 17, −24, −2
Right superior temporal gyrus	54, −19, 1
Left superior temporal gyrus	−57, −20, 1
Right primary auditory cortex	50, −21, 7
Left primary auditory cortex	−52, −19, 7
Additional regions	
Right superior frontal gyrus	22, 26, 45
Left superior frontal gyrus	−22, 24, 44
Right medial frontal gyrus	2, 44, −6
Left medial frontal gyrus	−4, 48, 24

The Wake Forest University PickAtlas toolbox (version 3.0.5; Maldijan et al., 2014) was used to define these regions. Statistical significance level was set at *p* < 0.05 for FWE correction, consistent with the whole-brain analysis; TIV was included as a covariate of no interest. Analyses were conducted both with and without age being a covariate, to understand the impact of age.

### DTI Data Preprocessing and Analysis

#### Preprocessing

Diffusion-weighted imaging (DWI) data were acquired using the same 3.0 T Siemens Prisma MRI scanner, with the following parameters: orientation = sagittal, TR = 16,000 ms, TE = 98 ms, voxel size = 1.9 × 1.9 × 2.0 mm^3^, diffusion directions = 59, *b*-value 1 = 0 s/mm^2^, *b*-value 2 = 1000 s/mm^2^.

Raw DICOM files were converted to NIFTI format using MRIcon software (MRIcon, https://www.nitrc.org/projects/mricron/). Preprocessing was performed using the FMRIB Software Library (FSL https://fsl.fmrib.ox.ac.uk/fsl/fslwiki). Eddy-current-induced distortions were corrected using FSL’s eddy tool, followed by skull stripping with the BET (Brain Extraction Tool). Diffusion tensors were then fitted using the DTIFIT tool from FSL’s Diffusion Toolbox to generate fractional anisotropy (FA) maps for each participant.

#### DTI Analysis

DTI analysis followed procedures outlined in Khan et al. [35]. Group comparisons were conducted using Tract-Based Spatial Statistics (TBSS, Smith et al. 2006) implemented in the FSL. All FA images were aligned to a 1mm^3^ standard atlas space to ensure spatial consistency across participants. A mean FA skeleton was generated, and individual FA maps were projected onto this skeleton to isolate the centers of major white matter tracts. The randomize tool was used with 5000 iterations and threshold-free cluster enhancement (TFCE) to test group differences, chosen for its ability to enhance cluster detection without predefined thresholds. Based on the previous publication [35], only clusters of 100 or more contiguous voxels were considered when evaluating group differences.

For the group-level FA analysis, two-sample *t*-tests were conducted between the TIN and CON groups, both with and without age included as a covariate to assess the influence of age-related variability. To evaluate interactions between subgroups, additional *t*-tests were performed. Statistical significance was set at *p*-value < 0.05, corrected for multiple comparisons using FWE correction.

## Results

### Behavioral Results

Audiograms for each group and subgroup are shown in Fig. 1 to provide a hearing status context. These plots display group-averaged thresholds across standard and extended audiometric frequencies, offering a visual summary of hearing status.

Table 2 summarizes the demographic and behavioral characteristics of the main groups (TIN, CON) and subgroups (CON_NH_, CON_HL_, TIN_NH_, TIN_HL_).

**Table 2:** Demographic information about groups and subgroups

Group (*n*)	Age (years)	Gender	BAI	BDI-II	PTA in dB HL	HFPTA in dB HL	TFI
CON (28)	40.00 ± 9.86	22 M, 6F	0.64 ± 1.37	1.43 ± 2.77	12.50 ± 0.93*	13.90 ± 14.48	N/A
CON_NH_ (17)	38.71 ± 9.68	12 M, 5F	0.88 ± 1.58	2.06 ± 3.36	9.34 ± 0.35	8.19 ± 9.33	N/A
CON_HL_ (11)	42.0 ± 9.80	10 M, 1F	0.27 ± 0.90	0.45 ± 0.93	17.39 ± 2.61	22.73 ± 16.53	N/A
TIN (40)	46.68 ± 10.60*	34 M, 4 F, 2O	2.00 ± 3.88*	2.92 ± 4.41	20.71 ± 6.03*	24.95 ± 16.91*	27.18 ± 14.88
TIN_NH_ (15)	39.93 ± 8.53	12 M, 2 F, 1O	0.87 ± 1.25	2.80 ± 3.14	10.29 ± 0.34	8.22 ± 7.00	22.23 ± 13.89
TIN_HL_ (25)	**50.72 ± 9.62**† ‡	22 M, 2 F, 1O	2.68 ± 4.72	3.00 ± 5.11	26.95 ± 9.62	**41.67 ± 22.87**† ‡	32.16 ± 14.68

*CON*_*NH*_ controls with no hearing loss, *CON*_*HL*_ controls with hearing loss *TIN*_*NH*_ tinnitus patients with no hearing loss, *TIN*_*HL*_ tinnitus patients with hearing loss, *PTA* pure-tone average of 0.5, 1, 2, 4 kHz in both ears, *HFPTA* high-frequency pure-tone average of 4, 6, and 8 kHz in both ears, *F* female, *M* male, *O* ‘Other’, *BAI* beck anxiety inventory, *BDI-II* beck depression inventory, *TFI* tinnitus functional index, *N/A* not applicable

Mean ± standard deviation for participants’ demographics. * significant difference between TIN and CON groups. † significant difference between TIN_HL_ and CON_NH_ groups. ‡ significant difference between TIN_HL_ and TIN_NH_ groups. With a significance level of 0.05

Demographic comparisons revealed a significant age difference between the groups. A one-way ANOVA comparing the subgroups also indicated a significant age effect (*F* = 6.69, *p* < 0.001). Post-hoc Tukey HSD test confirmed that participants in the TIN_HL_ subgroup were significantly older than those in the CON_NH_, CON_HL,_ and TINN_H_ (all *p* < 0.05). No significant group differences were observed in gender distribution (*χ*^2^ = 0.473, *p* = 0.491).

Behavioral comparisons showed that the TIN group had significantly higher Beck Anxiety Inventory (BAI) scores than the CON group (*t* = 2.57, *p* = 0.013), and this remained significant after adjusting for multiple comparisons. Subgroup comparisons did not reveal any significant differences in BAI scores (*F* = 2.43, *p* = 0.083). No significant group differences were found in Beck Depression Inventory-II (BDI-II) scores at either the group (*t* = 1.54, *p* = 0.129) or subgroup level (*F* = 1.24, *p* = 0.3).

Audiometric comparisons revealed that the TIN group exhibited significantly higher pure-tone average (PTA) thresholds (*t* = 3.94, *p* = 0.0002) and high-frequency PTA (HFPTA) thresholds (*t* = 2.68, *p* = 0.031) compared to the CON group; both remained significant after correction for multiple comparisons.

### VBM Results

#### Whole-Brain Analysis

Whole-brain VBM analysis, corrected for family-wise error (FWE), revealed no significant GM volume differences between the TIN and CON groups. Similarly, no significant differences were observed among the four subgroups (CON_NH_, CON_HL_, TIN_NH_, TIN_HL_) in relation to tinnitus or hearing loss.

#### Region of Interest (ROI) Analysis

**Fig. 2:**
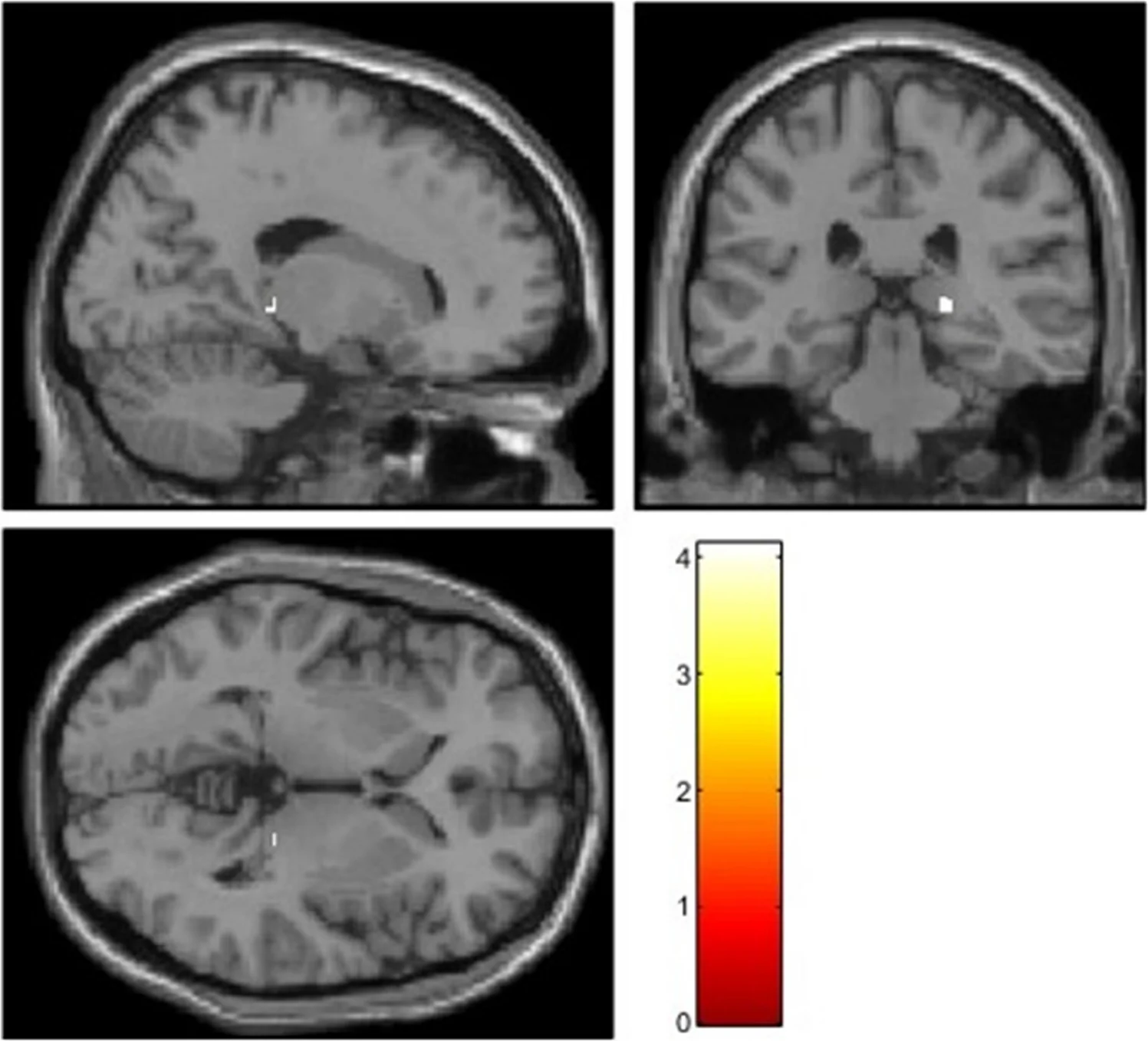
Region of significantly reduced gray matter volume in the thalamus proper (MNI coordinates: 21, −30, 0) associated with tinnitus and hearing loss. The highlighted region indicates decreased volume (*p* < 0.05, FWE-corrected). The color bar represents the t-value

ROI-based analysis, corrected for FWE, showed no significant differences between the TIN and CON groups overall. A small cluster in the thalamus (MNI coordinates: 21, −30, 0) shows lower volume in the TIN_HL_ group compared to the CON_NH_ group (*p* < 0.05, FWE-corrected; Fig. 2). This cluster was not significant after including age as a covariate.

#### Uncorrected Whole-Brain Analysis

Uncorrected whole-brain VBM results were examined to explore potential neuroanatomical patterns, given the absence of FWE-corrected effects. At an uncorrected p-value threshold of < 0.001, several clusters showed group differences in gray matter volume.

##### The Main Effect of Tinnitus

**Fig. 3:**
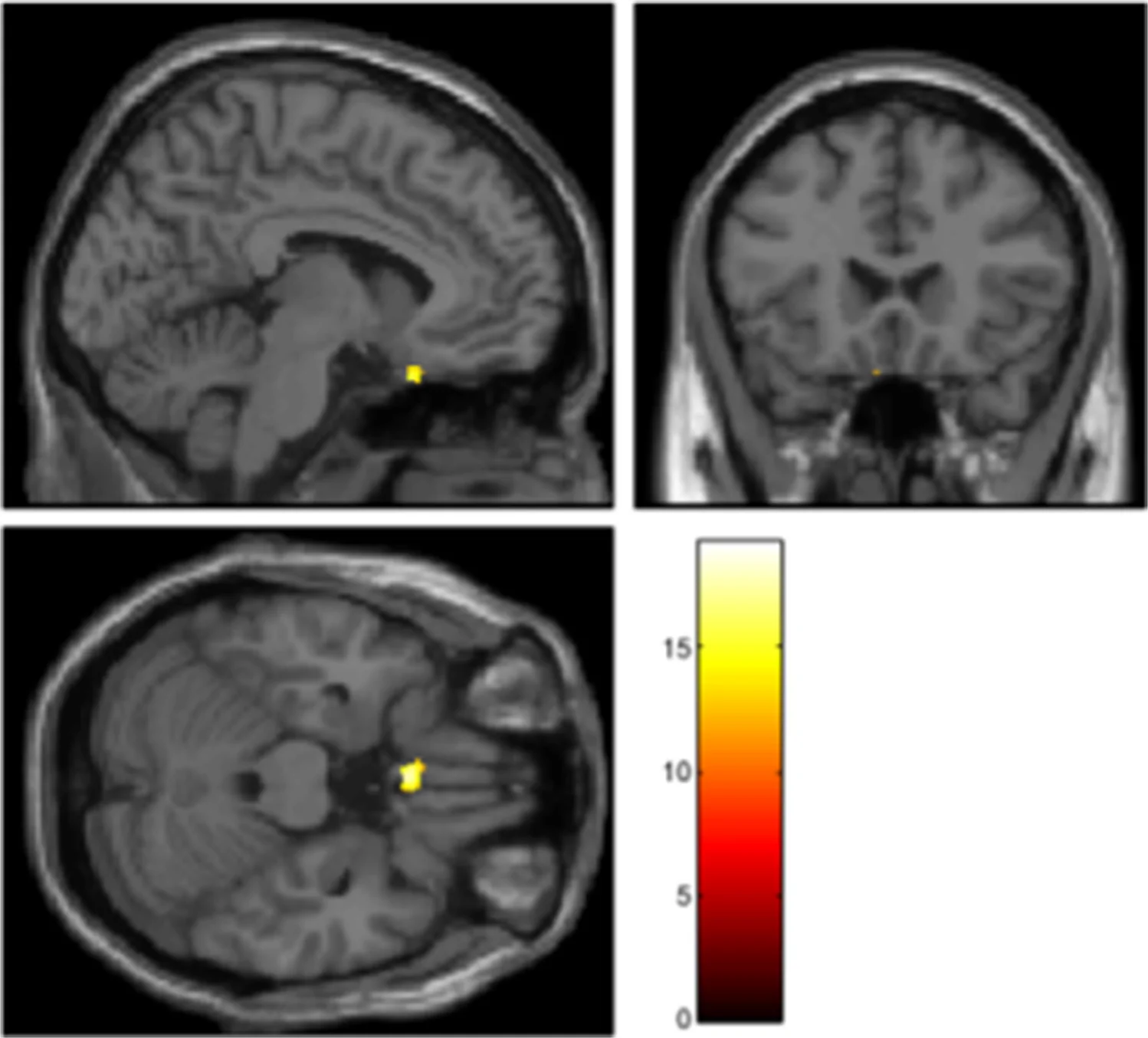
Region of significantly reduced gray matter in the right subcallosal area (MNI coordinates: 12, 9, −20) in the TIN group compared to the CON group. The highlighted area indicates decreased volume (*p* < 0.001, uncorrected), with an extent threshold of 50 voxels, after controlling for age. The color bar represents the *F*-value

At the uncorrected threshold of *p* < 0.001, the comparison between the TIN and CON groups showed decreased GM volume in the bilateral subcallosal areas associated with tinnitus. After controlling for age, only the right subcallosal area (MNI coordinates: 12, 4, −20) remained significant, as shown in Fig. 3. A similar uncorrected cluster was observed in the TIN_NHL_ < CON_NHL_ contrast.

##### The Main Effect of Hearing Loss

At the uncorrected threshold of *p* < 0.001, group differences related to hearing loss were observed in the contrasts TIN_HL_ < TIN_NH_ and CON_HL_ < CON_NH_. Clusters appearing in both contrasts included the left fusiform gyrus, right inferior frontal gyrus, left middle cingulate gyrus, right thalamus proper, left planum polare, right subcallosal area, left lingual gyrus, left inferior temporal gyrus, left cerebellum, right precentral gyrus, and left angular gyrus.

**Fig. 4:**
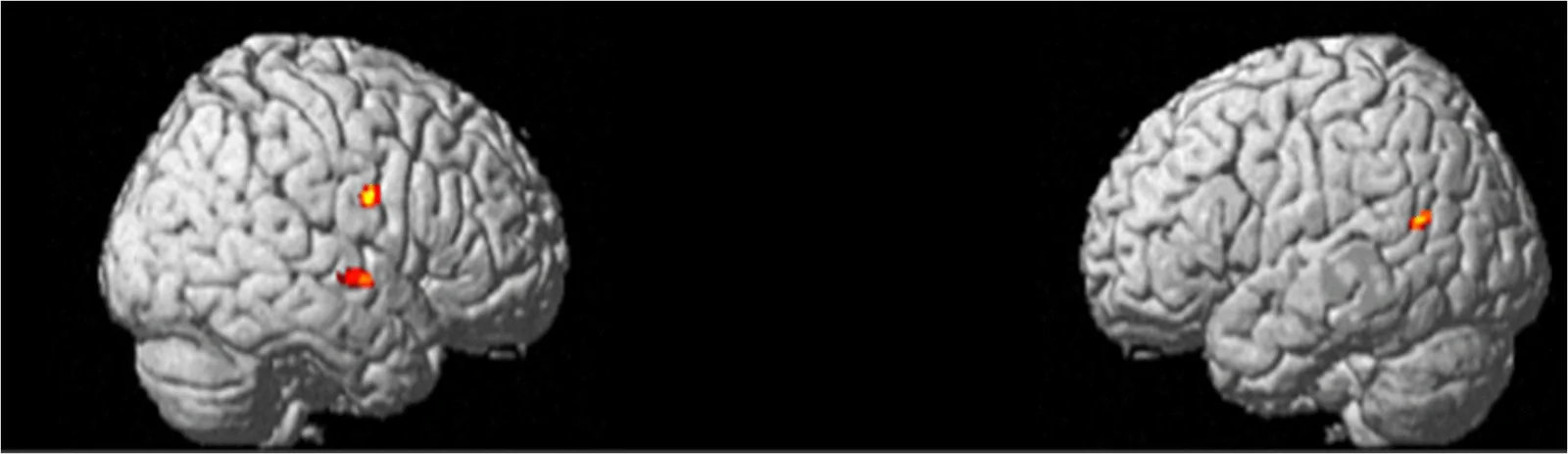
Brain regions showing significantly reduced gray matter volume in the **CON**_**HL**_ group compared to the **CON**_**NHL**_ group, after controlling for age. Highlighted areas (in red) include the right planum polare (MNI coordinates: 50, 9, −3), right precentral gyrus (MNI coordinates: 54, −3, −10), and left fusiform gyrus (MNI coordinates: −30, −54, −10). These regions showed decreased volume at uncorrected *p* < 0.001, with an extent threshold of 50 voxels

After including age as a covariate in the CON_HL_ < CON_NH_ contrast, clusters remained in the right planum polare (MNI coordinates: 50, 9, −3), right precentral gyrus (MNI coordinates: 54, −3, −10), and left fusiform gyrus (MNI coordinates: −30, −54, −10). These regions are illustrated in Fig. 4.

##### Combined Effect of Tinnitus and Hearing Loss

**Fig. 5:**
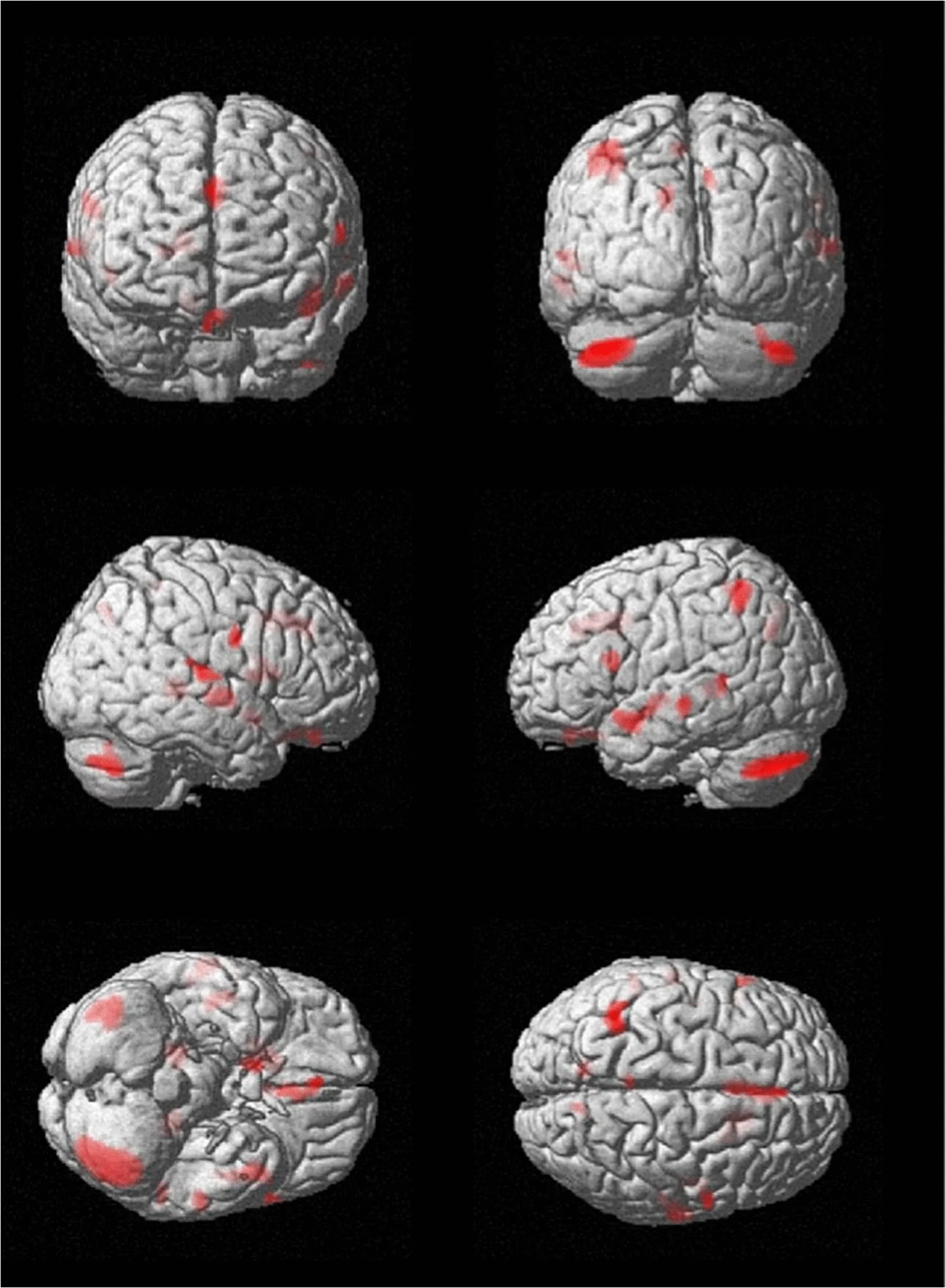
Brain regions with significantly reduced gray matter volume in the TIN_HL_ group compared to the CON_NH_ group, without controlling for age. Highlighted areas (in red) are shown in axial, coronal, and sagittal views and represent clusters identified at an uncorrected threshold of *p* <.001, with an extent threshold of 50 voxels; anatomical regions are defined in the main text above

At the uncorrected threshold *(p* < 0.001), the TIN_HL_ and CON_NH_ contrast showed reduced gray matter volume in several brain regions, suggesting a combined effect of tinnitus and hearing loss. These regions included the right caudate nucleus (MNI coordinates: 14, 14, 16), left temporal pole (MNI coordinates: −45, 6, −15), right superior frontal gyrus (MNI coordinates: 2, 26, 38), right hippocampus (MNI coordinates: 20, −38, 3), left superior parietal lobe (MNI coordinates: −38, −54, 56), left cerebellum (MNI coordinates: −42, −63, −40), right planum temporale (MNI coordinates: 63, −20, 9), right planum polare (MNI coordinates: 48, −8, −4), left precuneus (MNI coordinates: −12, −66, 34), left hippocampus (MNI coordinates: −18, −38, 2), right precuneus (MNI coordinates: 8, −72, 44), right subcallosal area (MNI coordinates: 14, 3, −12), left inferior frontal gyrus (MNI coordinates: −58, 15, 16), and another left precuneus location (MNI coordinates: −4, −44, 56). These clusters are illustrated in Fig. 5.

None of these clusters remained significant after controlling for age. Table 3 lists all reported brain regions from the whole-brain analysis at an uncorrected level (*p* < 0.001). Structures that remained significant after controlling for age are indicated in bold and marked with an asterisk “*”.

**Table 3:** VBM results on an uncorrected level

Contrast	Brain regions	MNI coordinates			*p*-uncorrected	Cluster size	*Z* score
		X	Y	Z		(k)	
Tinnitus							
TIN < CON TIN_NH_ < CON_NH_ TIN_HL_ < CON_HL_	Right subcallosal area* Subgenual anterior cingulate cortex Right subcallosal area Left subcallosal area Right subcallosal area* Left middle frontal gyrus Left subcallosal area	12 0 10 −10 12 −33 −16	9 18 9 12 4 63 10	−20 −26 −18 −15 −20 9 −16	< 0.001 < 0.001 < 0.001 < 0.001 < 0.001 < 0.001 < 0.001	77 107 321 109 63 70 92	3.36 3.91 3.87 3.31 3.60 3.55 3.50
Hearing loss							
TIN_HL_ < TIN_NH_ CON_NH_ > CON_HL_	Right fusiform gyrus Right inferior frontal gyrus Left middle cingulate gyrus Right thalamus proper Left planum polare Right planum polare Left lingual gyrus Left inferior temporal gyrus Left cerebellum Right subcallosal area Right planum polare* Right precentral gyrus* Left angular gyrus Left fusiform gyrus*	32 54 0 15 −46 45 −22 −22 −44 28 50 54 −58 −30	−56 20 3 −32 −10 −8 −66 −2 −64 9 −9 −3 −60 −54	−10 22 32 −4 2 −10 −6 −46 −28 10 −3 24 18 −10	< 0.001 < 0.001 < 0.001 < 0.001 < 0.001 < 0.001 < 0.001 < 0.001 < 0.001 < 0.001 < 0.001 < 0.001 < 0.001 < 0.001	120 202 669 265 445 187 95 55 267 105 117 70 54 111	4.45 4.19 4.18 4.13 3.92 3.90 3.84 3.71 3.62 3.47 4.01 4.09 3.78 3.54
Tinnitus and hearing loss							
TIN_HL_ < CON_NH_	Right caudate nucleus Left temporal pole Right superior frontal gyrus Right hippocampus Left superior parietal lobe Left cerebellum Right planum temporale Right planum polare Left precuneus Left hippocampus Right precuneus Right subcallosal area Left inferior frontal gyrus Left precuneus	14 −45 2 20 −38 −42 63 48 −12 −18 8 14 −58 −4	14 6 26 −38 −54 −63 −20 −8 −66 −38 −72 3 15 −44	16 −15 38 3 56 −40 9 −4 34 2 44 −12 16 56	< 0.001 < 0.001 < 0.001 < 0.001 < 0.001 < 0.001 < 0.001 < 0.001 < 0.001 < 0.001 < 0.001 < 0.001 < 0.001 < 0.001	315 462 600 331 476 1294 195 244 118 208 50 166 68 51	4.42 4.34 4.21 4.51 4.08 4.01 3.94 3.94 3.85 3.70 3.89 3.59 3.57 3.57

Regions in bold and marked with an asterisk “*” remained significant after controlling for age

### DTI Results

#### Main Effect of Tinnitus

Significantly lower fractional anisotropy (FA) values were observed in the TIN group compared to the CON group in several white matter tracts. These included the left anterior thalamic radiation, genu of the corpus callosum, superior longitudinal fasciculus, the body of the corpus callosum, right inferior longitudinal fasciculus, forceps major, posterior corona radiata, inferior fronto-occipital fasciculus, and inferior longitudinal fasciculus.

**Fig. 6:**
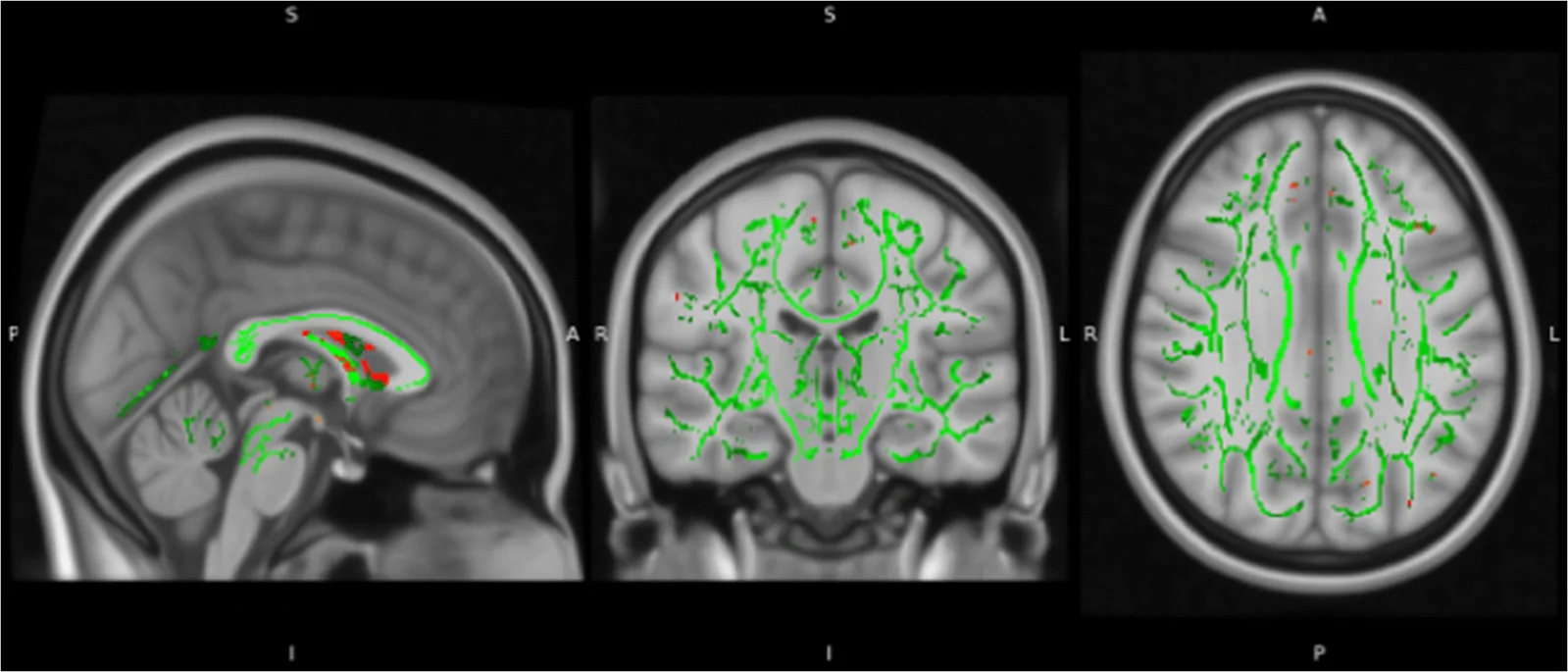
White matter regions showing significantly reduced FA in the TIN group compared to the CON group, without controlling for age. Green lines represent major white matter tracts, while red clusters highlight significant findings (*p* < 0.05). Highlighted clusters are in the following tracts: the left anterior thalamic radiation, genu and body of corpus callosum, superior longitudinal fasciculus, right inferior fronto-occipital fasciculus, and inferior longitudinal fasciculus, with the largest clusters in the superior longitudinal fasciculus (17, 29, 50) and corticospinal tract (3, −56, −19)

However, after including age as a covariate of no interest, none of these clusters remained significant. Figure 6 illustrates the white matter regions showing significant FA reductions in the TIN group before controlling for age, with the largest coherent clusters in the superior longitudinal fasciculus (17, 29, 50) and corticospinal tract (3, −56, −19).

#### The Main Effect of Hearing Loss

Before controlling for age, significantly reduced FA values were observed in the CON_HL_ group compared to the CON_NH_ group in the following white matter tracts: right anterior thalamic radiation, left and right inferior fronto-occipital fasciculus, left and right superior longitudinal fasciculus, right anterior corona radiata, right forceps minor, splenium of the corpus callosum, and right corticospinal tract.

**Fig. 7:**
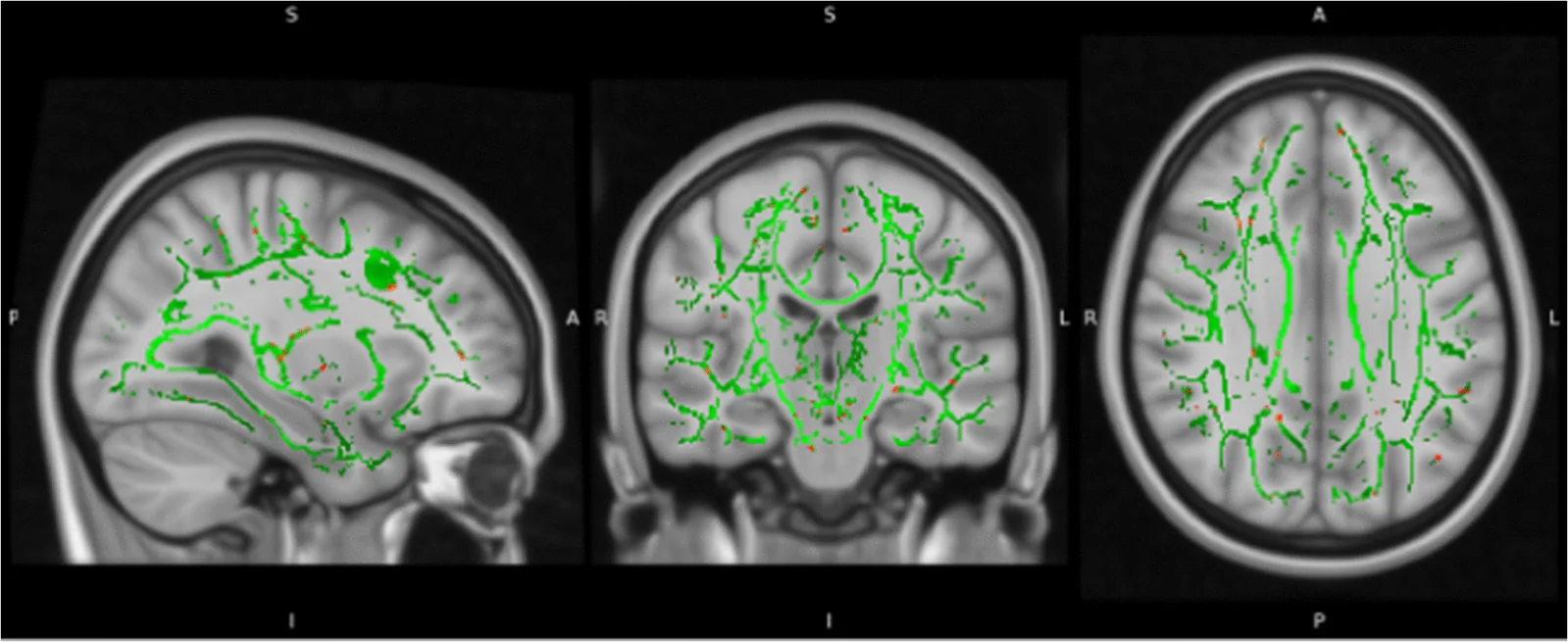
Sagittal, coronal, and axial views showing white matter regions with significantly reduced FA in the CON_NH_ group compared to the CON_HL_ group, after controlling for age. Green lines represent major white matter tracts, while red clusters highlight significant findings (*p* < 0.05). Significant regions include the right anterior thalamic radiation, the left superior longitudinal fasciculus, and the right corticospinal tract, with the largest coherent clusters located in the left superior longitudinal fasciculus (−31, 9, −2)

After including age as a covariate of no interest, significant reductions remained in the right anterior thalamic radiation, left superior longitudinal fasciculus, and right corticospinal tract, with the largest coherent clusters located in the left superior longitudinal fasciculus (−31, 9, −2). These findings are illustrated in Fig. 7.

#### The Combined Effect of Tinnitus and Hearing Loss

**Fig. 8:**
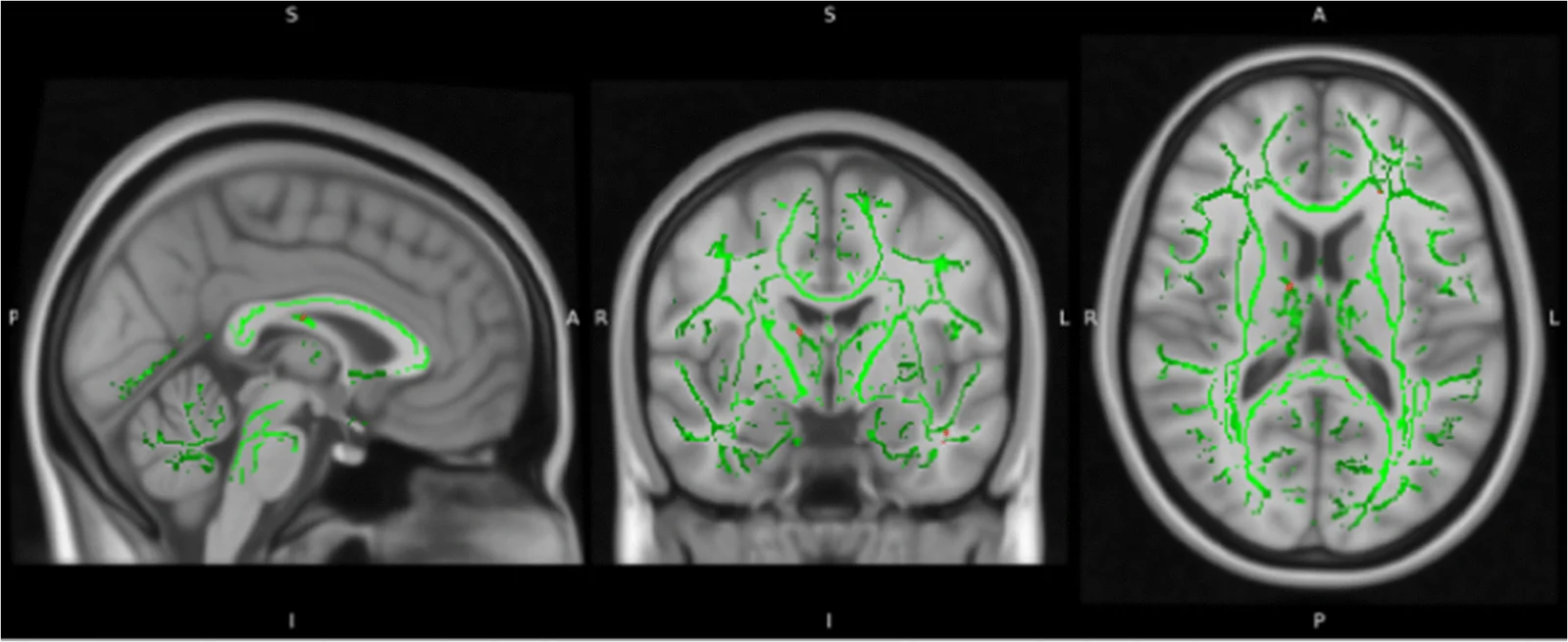
Sagittal, coronal, and axial views showing white matter regions with significantly reduced FA in the TIN_HL_ group compared to the CON_NH_ group, after controlling for age. Green lines represent white matter tracts, while red and yellow areas highlight significant findings (*p* < 0.05). Significant regions include the forceps minor, right superior longitudinal fasciculus, left inferior longitudinal fasciculus, and anterior thalamic radiation, with the largest coherent clusters in the right superior longitudinal fasciculus (−18, 36, 29) followed by the forceps minor (−16, 30, 29)

The combined effect of tinnitus and hearing loss after controlling for age was associated with significantly reduced FA in the forceps minor, right superior longitudinal fasciculus, left inferior longitudinal fasciculus, and anterior thalamic radiation, with the largest coherent clusters in the right superior longitudinal fasciculus (−18, 36, 29) followed by the forceps minor (−16, 30, 29). These findings are illustrated in Fig. 8.

A summary of the results corrected for FWE (*p* < 0.05) is presented in Table 4. Additionally, Table 4 provides an overview of the key findings from both the VBM and DTI analyses Table 5.

**Table 4:** DTI results at the corrected level (*p* < 0.05, FWE-corrected); the different contrasts highlight white matter orientation differences between the groups

Contrast	Findings
TIN < CON	Left anterior thalamic radiation, genu of the corpus callosum, superior longitudinal fasciculus, the body of corpus callosum, right inferior longitudinal fasciculus, forceps major, posterior corona radiata, inferior fronto-occipital fasciculus, inferior longitudinal fasciculus
CON_NH_ > CON_HL_	Right anterior thalamic radiation*, left superior longitudinal fasciculus*, right corticospinal tract *
CON_NH_ > TIN_HL_	Forceps minor*, right superior longitudinal fasciculus*, left inferior longitudinal*, anterior thalamic radiation*

Regions in bold and marked with an asterisk “*” remained significant after controlling for age

**Table 5:** Summary of the most important findings from VBM and DTI analyses

Contrast	VBM findings	DTI findings
TIN < CON	Whole-brain analysis (uncorrected *p* < 0.001): Right subcallosal area* Subgenual anterior cingulate cortex Right subcallosal area Left subcallosal area	(FWE-corrected * p* < 0.05): Left anterior thalamic radiation Genu of the corpus callosum Superior longitudinal fasciculus (FWE-corrected * p* < 0.05): The body of the corpus callosum Right inferior longitudinal fasciculus Forceps major Posterior corona radiata Inferior fronto-occipital fasciculus Inferior longitudinal fasciculus
CON_HL_ < CON_NH_	Whole-brain analysis (uncorrected * p* < 0.001): Right planum polare* Right precentral gyrus* Left fusiform gyrus* Left angular gyrus	(FWE-corrected * p* < 0.05): Right anterior thalamic radiation* Left and right superior longitudinal fasciculus* Left and right Inferior longitudinal fasciculus* Forceps major* Body of the corpus callosum*
TIN_HL_ < CON_NH_	ROI (FWE-corrected * p* < 0.05): Thalamus Whole-brain analysis (uncorrected * p* < 0.001): Right caudate nucleus Left temporal pole Right superior frontal gyrus Right hippocampus Left superior parietal lobe Left cerebellum Right planum temporale Right planum polare Left precuneus Left hippocampus Right precuneus Right subcallosal area Left inferior frontal gyrus Left precuneus	(FWE-corrected * p* < 0.05): Forceps minor* Right superior longitudinal fasciculus* Left inferior longitudinal fasiculus* Anterior thalamic radiation*

This table summarizes significant findings from VBM and DTI analyses across group contrasts. Regions in bold and marked with an asterisk “*” remained significant after controlling for age

### Effect Sizes in VBM and DTI Studies

To better understand the significance or lack thereof of our results, we conducted an exploratory analysis of the effect sizes of our findings, which are descriptive and hypothesis-generating rather than a basis for reinterpreting non-significant results. Effect sizes for both VBM and DTI comparisons between tinnitus and control groups were calculated using G*Power and are presented in Supplementary Tables 1–3.

For the VBM whole-brain analysis, effect sizes were generally very small to small, ranging from Cohen’s *d* = 0.02 to 0.1, depending on the comparison. The CON_HL_ vs CON_NH_ comparison showed the largest effect size.

ROI-level VBM analyses showed slightly stronger effect sizes than whole-brain, ranging from very small (*d* = 0.02) to medium (*d* = 0.58). Again, the CON_HL_ vs CON_NH_ comparison produced the largest effect.

In contrast, DTI analyses revealed multiple large effect sizes, particularly in comparisons among tinnitus and control participants. Cohen’s *d* ranged from *0.2* in CON_HL_ vs CON_NH_ to *d* = *0.98* in the TIN_HL_ vs CON_HL_ comparison, which showed the largest effect.

For comparison, effect sizes were also estimated using previously published civilian data from our lab [20, 36]. These effects were generally smaller and required substantially larger samples to achieve comparable power, compared to the present findings. However, the trend of DTI yielding stronger effects than VBM remained. Full results from these civilian comparisons are presented in Supplementary Tables 4–6.

## Discussion

This study aimed to investigate neuroanatomical changes in gray and white matter in a military population. Our study is the first to examine neuroanatomical differences in military personnel with tinnitus and hearing loss, a population uniquely exposed to impulse noise and blast trauma. Our results indicated that in gray matter, statistically significant findings emerged only when examining the combined effects of tinnitus and hearing loss using a hypothesis-driven approach, with an auditory region mask. All other findings concerning gray matter were significant only at the uncorrected level. The absence of robust GM differences (whole-brain Cohen’s *d* < 0.15, ROI-level effects ranging up to medium *d* = 0.58) highlights that GM alterations were subtle. However, the ROI-level effects suggest that VBM is better suited for detecting hearing-loss-related changes with adequate sample sizes.

In contrast, the DTI analysis revealed more robust and widespread effects of tinnitus, hearing loss, and their combination on white matter, with significant findings across all conditions. These results suggest that DTI may be a more sensitive tool to assess tinnitus-related alterations than VBM. This pattern was also reflected in our exploratory analysis of effect-size estimates, which ranged from medium (*d* = 0.4) to large WM effects (up to *d *= 0.98). Taken together, these findings suggest that DTI may be especially suited for examining tinnitus-related changes, while VBM may be more informative for hearing-loss-related structural differences, reflecting modality-specific sensitivities rather than exclusive effects.

### VBM

#### Role of the Thalamus in Tinnitus and Hearing Loss

The only statistically significant finding at the FWE-corrected level in our analysis was observed in the ROI contrast between CON_NH_ > TIN_HL_, specifically in the right thalamus. Notably, this effect was only present when age was not included as a covariate of no interest.

Muhlau et al. [16] previously found increased thalamic gray matter volume in people with tinnitus relative to healthy controls. However, unlike our study, their participants had normal hearing, which may explain the discrepancy, as our findings highlight differences specific to hearing loss groups that were not observed in their normal-hearing cohort. The presence of additional hearing loss in our cohort could be a key factor influencing thalamic morphology.

Supporting this, Tae et al. [44] observed increased gray matter volume in the left thalamus and decreased volume in the right thalamus in patients who had both tinnitus and mild hearing loss compared to healthy controls. The inconsistencies across studies may be attributed to differences in cohort characteristics, including participant age, tinnitus duration, degree of hearing loss, and comorbid conditions. For example, Muhlau et al. [16] and Tae et al. [44] reported varying hearing status across their samples, which may account for inconsistent findings. However, only a few VBM studies have reported thalamic volume; a substantial body of functional MRI research has demonstrated reduced connectivity between the thalamus, the medial geniculate body (MGB), and cortical regions in individuals with tinnitus, often in the context of co-occurring hearing loss [45–48].

Our finding of decreased gray matter volume in the thalamus in patients with both tinnitus and hearing loss, especially near the MGB, aligns with two prominent theoretical models of tinnitus. The thalamocortical dysrhythmia model [49] posits that hearing loss leads to abnormal low-frequency oscillations in the thalamus, disrupting normal thalamocortical rhythms and resulting in auditory cortex hyperactivity perceived as tinnitus. Alternatively, the noise-cancellation hypothesis [50] suggests that failure of the MGB to suppress irrelevant auditory signals leads to the conscious perception of phantom sounds. Both models emphasize the central role of the MGB in tinnitus pathophysiology and may account for the structural changes observed in our study.

#### Limbic and Default Mode Network Contributions to Tinnitus

At the uncorrected level, we observed decreased gray matter volume in the right subcallosal area and the precuneus. Although these findings did not survive correction for multiple comparisons, both regions have been consistently implicated in tinnitus pathophysiology. The subcallosal area, located near the subgenual anterior cortex and nucleus accumbens, has been linked to inhibitory feedback and emotional regulation [16, 17, 19, 51], while the precuneus, a central hub of the default network, has been repeatedly associated with persistent awareness of tinnitus [22, 52, 53]. Together, these regions highlight the potential involvement of limbic-DMN interactions in tinnitus, particularly in the emotional salience and attentional awareness of phantom auditory percepts.

### DTI

Our DTI results indicated that, when controlling for age, hearing loss is associated with more widespread and statistically robust reductions in white matter integrity than tinnitus. Significant FA decreases were observed in the right anterior thalamic radiation, bilateral superior longitudinal fasciculus, bilateral inferior longitudinal fasciculus, forceps major, body of corpus callosum, and right corticospinal tract, all of which remained significant after controlling for participant age. These findings are consistent with previous research [15, 35, 54–56], which suggests that auditory deprivation leads to diffuse structural reorganization across sensory and cognitive pathways.

At the same time, our exploratory effect size analysis revealed that tinnitus-related contrasts consistently produced larger effect sizes than hearing-loss-only contrasts, despite failing to survive correction when age was included. This pattern illustrates the distinction between the magnitude of group differences and their statistical reliability. Tinnitus may exert stronger localized impacts on specific tracts, but limited sample size, variance, and overlap with age-related changes diffuse FA statistical power. In contrast, hearing loss produced smaller but more spread FA reductions that were consistent enough across participants to remain significant after correction.

Interestingly, when tinnitus co-occurred with hearing loss, the reductions in FA were sometimes less pronounced than in individuals with hearing loss alone. In other words, white matter integrity appeared somewhat better preserved in the combined group. This pattern suggests that tinnitus may alter the trajectory of hearing-loss-related white matter changes, potentially by engaging compensatory neural mechanisms rather than simply amplifying structural decline.

A related methodological challenge involves the use of age as a covariate. While controlling for age accounts for age-related brain changes, it may also obscure tinnitus-related effects on white matter integrity [57], given the interrelationship between age and tinnitus, as was similarly observed in our gray matter analysis. This overlap in affected brain regions between tinnitus and aging, particularly in limbic and auditory regions, complicates interpretation and highlights the need for careful covariate selection in neuroimaging studies.

Functionally, the affected tracts span multiple domains: attention and executive control (anterior thalamic radiation, superior longitudinal fasciculus), hemispheric communication and multimodal integration (corpus callosum, forceps major, inferior fronto-occipital fasciculus), and sensorimotor/visual-auditory pathways (corticospinal tract, inferior longitudinal fasciculus, posterior corona radiata). Supporting this, a recent paper by Xu et al. [38] reported white matter changes due to tinnitus in the forceps minor and right corticospinal tract, overlapping with our findings. Notably, their study found that white matter changes were more evident than gray matter changes, which again aligns with our observation.

Based on these results, we propose that tinnitus is not associated with a discrete “neural signature” in white matter. Instead, it involves widespread alterations across multiple tracts, reflecting its complex, network-based nature that integrates auditory, cognitive, and emotional systems.

### Combined VBM and DTI

The thalamus emerged as the only overlapping region identified in both VBM and DTI analyses, consistent with prior work suggesting that hearing loss exerts a strong influence on thalamic structure [15]. This overlap underscores the thalamus as a potential hub where auditory deprivation impacts both gray and white matter pathways.

More broadly, our findings highlight the difficulty of separating the effects of tinnitus from those of hearing loss and aging. These factors are deeply intertwined and appear to exhibit simultaneous influences on both gray and white matter neuroanatomy. This complexity highlights the need for future studies to carefully control for and examine the interactive effects of these variables.

A central methodological issue concerns whether to control for age in VBM and DTI analyses. On one hand, gray matter volume and white matter integrity naturally decline with age [58], and including age as a covariate reduces confounds, enhances reproducibility, and improves comparability across studies. On the other hand, both tinnitus and hearing loss become more prevalent with age, meaning that controlling for age may inadvertently obscure genuine structural differences. Moreover, statistical models often assume a linear relationship between age and brain structure, which may not accurately reflect the nature of brain aging [59]. This dilemma is particularly relevant in studies with modest sample sizes, where correcting for age can reduce the statistical power and risk of overcorrection. In our military cohort, the issue is further complicated by unique noise exposures that were not directly assessed but may contribute to distinct neuroanatomical profiles compared to civilian populations.

### Effect Sizes

Effect sizes were calculated using standard metrics (Cohen’s *d* and Hedges’ *g*) based on group-level comparisons, as detailed in the Supplementary Methods. These exploratory analyses should be interpreted cautiously, serving primarily to guide planning for appropriately powered group sizes.

Whole-brain VBM analyses revealed the smallest effect sizes, suggesting only subtle group differences at the global level. ROI-based VBM analyses yielded somewhat stronger effects, highlighting the value of hypothesis-driven region selection for enhancing statistical power.

In contrast, DTI analyses revealed multiple medium-to-large effects, particularly in comparisons between tinnitus participants and controls. This pattern suggests that DTI may be more sensitive to tinnitus-related alterations, whereas VBM appears more closely associated with hearing-loss-related changes, with effect sizes providing descriptive context.

Notably, effect sizes tended to be larger in the military sample than in previously published civilian datasets [19, 35]. This may reflect reduced variability in tinnitus etiology, age range, and occupational context. Such relative homogeneity, combined with elevated risk, may enhance sensitivity to structural brain differences, suggesting that military populations could serve as a useful model for isolating neural mechanisms that are harder to detect in more heterogeneous civilian samples.

### Study Limitations and Future Directions

Whole-brain analysis did not appear sensitive enough to detect GM volume differences between tinnitus participants and controls, and no significant differences emerged at the subgroup level. The only significant finding from our VBM findings was lower GM volume in the thalamus, observed in participants with both tinnitus and hearing loss compared to controls. However, the effect size was very small, raising concerns about replicability and contributing to the variability often reported in VBM studies of tinnitus. Further investigations of GM changes should consider ROI approaches guided by prior hypotheses to enhance statistical power.

Importantly, structural brain changes in tinnitus may evolve gradually over time. Longitudinal designs may therefore be better suited to detect these dynamics, particularly in chronic or progressive cases. For example, Husain et al. [60] demonstrated intervention-related increases in GM volume using VBM, while Du et al. [61] identified progressive WM decreases using longitudinal DTI in tinnitus patients with sleep disturbance. These findings emphasize the limitations of cross-sectional designs, which may miss subtle or cumulative neuroplastic changes.

Although our DTI sample size fell short of calculated requirements, the observed effect sizes were larger than in VBM, and the corresponding sample sizes are more attainable for future studies. We encourage other researchers to replicate these findings and further explore WM differences that may distinguish tinnitus from controls. While identifying a single biomarker using FA may be challenging, multi-tract identification approaches, potentially supported by machine learning, may offer a more promising path forward.

Finally, while our study has several strengths, including the novelty of examining a military cohort and the inclusion of effect size estimates, we also acknowledge limitations such as small sample size, unequal subgroup distribution, and the specificity of our sample. The military population is characterized by a younger age distribution than many civilian studies, a higher proportion of males, and potentially unique neuroanatomical features due to occupational noise exposure. Because of this male-dominant composition, the study was not powered to examine sex-based or gender-based differences. These factors may limit generalizability but also provide a valuable opportunity to identify WM pathways most affected in military tinnitus and hearing loss, while recognizing that other structural features may also contribute to informing the development of imaging-based markers for diagnosis and rehabilitation in service members.

## Conclusion

In the understudied military cohort without comorbid psychiatric or neurological disorders, we examined both gray and white matter correlates of tinnitus and hearing loss, an approach that is not commonly used. VBM revealed only minimal and potentially non-replicable gray matter differences, whereas DTI uncovered widespread alterations across multiple white matter tracts. This contrast likely reflects the nature of tinnitus as a condition involving subtle microstructural differences across distributed brain regions, which DTI is better suited to detect than VBM. Our exploratory effect size findings were consistent with this pattern, with DTI yielding larger effects and correspondingly more attainable sample size estimates for future studies than VBM. Our findings support the idea that tinnitus is a complex, network-level condition involving auditory, cognitive, and emotional systems. While white matter effects appear more robust, they are not exclusive, as gray matter alterations were also observed. Together, these results highlight potential targets for future research.

## Supplementary Information

Below is the link to the electronic supplementary material.

ESM 1 (DOCX 24.1 KB)

## Data Availability

The data that support the findings of this study are available on request from the corresponding author, Dr. Fatima T Husain.

## Declarations

### Use of AI-Assited Technologies

In preparing this manuscript, the authors used Microsoft Copilot (Microsoft Corporation, Redmond, WA, USA; used in 2026) to support improvements in grammar, clarity, and readability. The tool was not involved in generating scientific content, conducting analyses, interpreting results, nor generating figures. The authors remain fully responsible for the accuracy and integrity of the manuscript.
