# Supplementary material for: White Matter Alterations Are More Robust Than Gray Matter Differences in Tinnitus and Hearing Loss: An MRI Study of Military Personnel
Source: J Assoc Res Otolaryngol. 2026 May 13;27(4):611–29. doi: 10.1007/s10162-026-01052-0 (PMC13504148; doi:10.1007/s10162-026-01052-0)
Supplement: Supplementary file 1 [file 10162_2026_1052_MOESM1_ESM.docx]

Supplementary Materials

Supplementary Methods: Effect Size Calculations

Effect sizes were calculated to complement statistical significance testing and assess the magnitude of group differences in brain structures. While p-values indicate whether an observed effect is unlikely to have occurred by chance, effect sizes provide information about the practical relevance of those differences, particularly in neuroimaging, where subtle structural changes may reflect meaningful neural alterations.

Effect sizes are commonly reported using Cohen’s d and Hedges’g, with the latter incorporating a correction for small sample sizes. Both metrics were calculated using group means and pooled standard deviation. According to Cohen’s guidelines [62, 63], values of 0.20, 0.50, and 0.80 represent small, medium, and large effect sizes, respectively. Estimated sample size- per groups were calculated using G*Power.

For the VBM whole-brain analysis, mean gray matter volume was extracted for each participant. Group means and standard deviations were then computed for the TIN and CON groups, as well as subgroups (TIN_HL_, TIN_NHL_, CON_HL_, CON_NHL_). For the ROI mask, the mean gray matter volume was extracted separately. Specifically, to estimate effect sizes for the auditory mask defined by Muhlau et al. [16], we extracted the volume of the regions included in the mask. For the white matter analysis, previously extracted FA values were averaged across each subgroup to facilitate group-level comparisons of effect sizes.

Supplementary Table 1. Effect sizes in Whole-Brain Analysis

| Contrast | Cohen’s d | Hedges’ g | Effect size | Estimated sample size -per group |
| --- | --- | --- | --- | --- |
| TIN HL vs TIN NH | -0.0224 | -0.022 | Very small effect | 31,287 |
| TIN HL vs CON HL | -0.1235 | -0.1208 | Small effect | 1,031 |
| TIN HL vs CON NH | 0.023 | 0.0226 | Very small effect | 29,676 |
| TIN NH vs CON HL | -0.113 | -0.1094 | Small effect | 1,231 |
| TIN NH vs CON NH | 0.0271 | 0.0264 | Very small effect | 21,376 |
| CON HL vs CON NH | 0.1385 | 0.1345 | Small effect | 820 |
| TIN vs CON | 0.0415 | 0.0404 | Very small effect | 9,116 |

The effect sizes and estimated needed sample sizes per group for ROI analysis are presented in Supplementary Table 2.

Supplementary Table 2. Auditory mask Muhlau et al. [16]

| Contrast | Cohen’s d | Hedges’ g | Effect size | Estimated needed n -per group |
| --- | --- | --- | --- | --- |
| TIN HL vs TIN NH | 0.024 | 0.023 | Very small effect | 27,254 |
| TIN HL vs CON HL | -0.249 | -0.243 | Small effect | 255 |
| TIN HL vs CON NH | 0.273 | 0.268 | Small effect | 212 |
| TIN NH vs CON HL | -0.305 | -0.295 | Small effect | 170 |
| TIN NH vs CON NH | 0.284 | 0.277 | Small effect | 196 |
| CON HL vs CON NH | 0.586 | 0.569 | Medium effect | 47 |
| TIN vs CON | 0.054 | 0.0529 | Very small effect | 5,385 |

The effect sizes and estimated needed sample sizes per group for DTI analysis are presented in Supplementary Table 3.

Supplementary Table 3. Effect sizes in DTI analysis

| Contrast | Cohen’s d | Hedges’ g | Effect size | Estimated needed n -per group |
| --- | --- | --- | --- | --- |
| TIN HL vs TIN NH | -0.4 | -0.4 | Small effect | 100 |
| TIN HL vs CON HL | 0.98 | 0.96 | Large effect | 18 |
| TIN HL vs CON NH | 0.78 | 0.77 | Medium effect | 27 |
| TIN NH vs CON HL | 0.88 | 0.85 | Large effect | 22 |
| TIN NH vs CON NH | 0.81 | 0.79 | Large effect | 25 |
| CON HL vs CON NH | 0.21 | -0.2 | Small effect | 357 |
| TIN vs CON | 0.8 | 0.7896 | Large Effect | 26 |

Supplementary Table 4. Effect sizes in Whole-Brain Analysis in the civilian population [20]

| Contrast | Cohen’s d | Hedges’ g | Effect size | Estimated needed n -per group |
| --- | --- | --- | --- | --- |
| TIN HL vs TIN NH | -0.285 | -0.278 | Small effect | 195 |
| TIN HL vs CON HL | 0.037 | 0.036 | Very small effect | 11,468 |
| TIN HL vs CON NH | 0.082 | 0.080 | Small effect | 2336 |
| TIN NH vs CON HL | 0.204 | 0.199 | Small effect | 379 |
| TIN NH vs CON NH | 0.360 | 0.351 | Small effect | 123 |
| CON HL vs CON NH | 0.011 | 0.011 | Very small effect | 129,735 |
| TIN vs CON | 0.112 | 0.109 | Small effect | 1253 |

Supplementary Table 5. Auditory mask Muhlau et al. [16] in the civilian population Kim et al. [20]

| Contrast | Cohen’s d | Hedges’ g | Effect size | Estimated needed n -per group |
| --- | --- | --- | --- | --- |
| TIN HL vs TIN NH | -0.461 | -0.449 | Small effect | 75 |
| TIN HL vs CON HL | 0.012 | 0.012 | Very small effect | 109,013 |
| TIN HL vs CON NH | -0.270 | -0.263 | Small effect | 217 |
| TIN NH vs CON HL | 0.313 | 0.305 | Small effect | 162 |
| TIN NH vs CON NH | 0.241 | 0.235 | Small effect | 272 |
| CON HL vs CON NH | -0.176 | -0.171 | Small effect | 508 |
| TIN vs CON | 0.010 | 0.010 | Very small effect | 156,979 |

Supplementary Table 6. Effect sizes in DTI analysis in the civilian population Khan et al. [36]

| Contrast | Cohen’s d | Hedges’ g | Effect size | Estimated needed n -per group |
| --- | --- | --- | --- | --- |
| TIN HL vs TIN NH | -0.678 | -0.674 | Medium | 36 |
| TIN HL vs CON HL | -0.346 | -0.344 | Small | 133 |
| TIN HL vs CON NH | -0.713 | -0.709 | Medium | 32 |
| TIN NH vs CON HL | 0.404 | 0.402 | Small | 98 |
| TIN NH vs CON NH | -0.085 | -0.085 | Very Small | 2,174 |
| CON HL vs CON NH | -0.457 | -0.454 | Medium | 77 |
| TIN vs CON | -0.372 | -0.370 | Small | 115 |
